# Distal Symmetric Polyneuropathy Identification in Type 2 Diabetes Subjects: A Random Forest Approach

**DOI:** 10.3390/healthcare9020138

**Published:** 2021-02-01

**Authors:** Valeria Maeda-Gutiérrez, Carlos E. Galván-Tejada, Miguel Cruz, Adan Valladares-Salgado, Jorge I. Galván-Tejada, Hamurabi Gamboa-Rosales, Alejandra García-Hernández, Huizilopoztli Luna-García, Irma Gonzalez-Curiel, Mónica Martínez-Acuña

**Affiliations:** 1 Unidad Académica de Ingeniería Eléctrica, Universidad Autónoma de Zacatecas, Jardín Juarez 147, Centro, 98000 Zacatecas, Zac, Mexico; valeria.maeda@uaz.edu.mx (V.M.-G.); gatejo@uaz.edu.mx (J.I.G.-T.); hamurabigr@uaz.edu.mx (H.G.-R.); alegarcia@uaz.edu.mx (A.G.-H.); hlugar@uaz.edu.mx (H.L.-G.); 2 Unidad de Investigación Médica en Bioquímica, Hospital de Especialidades, Centro Médico Nacional Siglo XXI. Instituto Mexicano del Seguro Social, Av. Cuauhtémoc 330, Col. Doctores, Del. Cuauhtémoc, Mexico City 06720, Mexico; miguel.cruzlo@imss.gob.mx (M.C.); adan.valladares@imss.gob.mx (A.V.-S.); 3 Unidad Académica de Ciencias Químicas, Universidad Autónoma de Zacatecas, Jardín Juarez 147, Centro, Zacatecas 98000, Mexico; irmacuriel@uaz.edu.mx (I.G.-C.); monicaimeldamtza@uaz.edu.mx (M.M.-A.)

**Keywords:** type 2 diabetes, distal symmetric polyneuropathy, feature selection, boruta, Random Forest

## Abstract

The prevalence of diabetes mellitus is increasing worldwide, causing health and economic implications. One of the principal microvascular complications of type 2 diabetes is Distal Symmetric Polyneuropathy (DSPN), affecting 42.6% of the population in Mexico. Therefore, the purpose of this study was to find out the predictors of this complication. The dataset contained a total number of 140 subjects, including clinical and paraclinical features. A multivariate analysis was constructed using Boruta as a feature selection method and Random Forest as a classification algorithm applying the strategy of K-Folds Cross Validation and Leave One Out Cross Validation. Then, the models were evaluated through a statistical analysis based on sensitivity, specificity, area under the curve (AUC) and receiving operating characteristic (ROC) curve. The results present significant values obtained by the model with this approach, presenting 67% of AUC with only three features as predictors. It is possible to conclude that this proposed methodology can classify patients with DSPN, obtaining a preliminary computer-aided diagnosis tool for the clinical area in helping to identify the diagnosis of DSPN.

## 1. Introduction

Diabetes Mellitus (DM), defined as a group of metabolic diseases characterized by hyperglycemia, resulting from defects in insulin secretion, action or both [1], is a multifactorial chronic disease that became a worldwide concern because of its epidemic proportions and complex management [2]. Especially, type 2 diabetes (T2D) because it is characterized by insulin resistance that induces organ dysfunction, and over 90% of DM are T2D [3]. Furthermore, T2D is associated with long-term complications (microvascular and macrovascular) involving tissue damage and organ failure. The most common microvascular complication is Diabetic Neuropathy (DN), because all types of diabetic patients insulin-dependent DM, non-insulin-dependent DM and, secondary diabetic patients can develop DN [4]. Diabetic neuropathies are a heterogeneous group of pathological manifestations with the potential to affect every organ with clinical implications, such as organ dysfunction which leads to low quality life and increased morbidity [5]. There are clinical classifications of DNs: symmetric and asymmetric. Distal Symmetrical Polyneuropathy (DSPN) which is the commonest type of DN, representing approximately 75% of cases [4]. DSPN is defined as peripheral nerve dysfunction with positive and negative symptoms and it is present in approximately 10% of recently diagnosed diabetic patients [5]. DSPN should not be diagnosed on the basis of one symptom, sign or test. In practice, the clinical area usually recommends five measures to be used in the diagnosis of DSPN [6]:

- Clinical measures
- Morphological and biochemical analyses
- Electrodiagnostic assessment
- Quantitative sensory testing
- Autonomic nervous system testing

This diagnosis include a family history of neuropathy particularly outside the context of diabetes, hammer toes, high arches, symptoms that slowly progress over many years, and neurologic examination abnormalities that are more pronounced than the patient’s symptoms [7]. Methods regarded as gold standards in clinical trials are not useful in clinical settings, because they are time consuming and require special devices [8] which are not common in all the public health services.

In Mexico, the prevalence of chronic complications in patients with T2D has increased. Sabag et al. [9] showed that DSPN affects 42.6% of the population. Furthermore, it is the complication that significantly impacts the quality of life of those affected.

In recent years, the research of diabetes care and the rapid advances in Artificial Intelligence (AI) has been a relevant topic. Four main categories aim to transform healthcare in the field of diabetes: Automated Retinal Screening, Clinical Decision Support, Predictive Population Risk Stratification, and Patient Self-Management Tools [10]. Due to this several studies have been using Machine Learning algorithms for the detection, identification, and monitoring of comorbidities such as neuropathy, nephropathy, wounds, and retinopathy. Alcalá-Rmz et al. [11] implemented an Artificial Neural Network (ANN), to determine if a patient presents diabetes based on a set of 19 para-clinical features. The model obtained statistically significant values with an AUC of 0.98 and an accuracy of 0.94. Moreover, Alcalá-Rmz et al. [12] proposed an implementation of Convolutional Neural Network (CNN) for classifying the four different stages of diabetic retinopathy using a total of 2644 images. The final model achieved an accuracy of 0.8065. Further, each class was evaluated under the statistic metric AUC: no diabetic retinopathy (0.79), mild (0.67), moderate (0.65), severe (0.69), and proliferative (0.79). In the work of Blobel et al. [13] it is proposed the implementation of Machine Learning (ML) methods, for early risk identification of diabetes polyneuropathy, based on structured electronic medical records. The dataset contains 238,590 laboratory records including episode identifiers, timestamp, varying number of measured parameters, laboratory test, retinopathy, nephropathy, age and, gender. The feature selection in this work was based on correlation analysis of the target class; the most significant features were glucose level in the blood and the urine. A comparison of five algorithms (Support Vector Machine [SVM], Decision Trees [DT], ANN, Linear Regression, and Logistic Regression[LR]) was done under different metrics. They concluded that ANN provides a better performance obtaining 89.88% of Area Under the Curve. Likewise, Metsker et al. [14] developed a structured procedure for predictive modeling, which includes data extraction, pre-processing, model adjustment, performance, and selection of the best models. The dataset comprises information about 5846 patients with diabetes. Finally, the models showed different results in terms of interpretation significance, Random Forest confirmed that the most important risk factor for polyneuropathy is the increased neutrophil level, on the other hand, linear models, showed linear dependencies of the presence of the disease on blood glucose levels and neural networks demonstrate the contribution of comorbidities to the development of polyneuropathy. Furthermore, Dagliati et al. [15] developed distinct models for microvascular complications, taking into account a temporal threshold for risk prediction of three, five, or seven years. They considered variables include demographic, clinical, and administrative data. The classification models used were LR, Näive Bayes (NB), SVM, and Random Forest. The feature selection was based on the Akaike information criterion. The validation of the results was in terms of Area Under the Curve, specifically, neuropathy demonstrated that Random Forest and SVM obtained the best performance when the datasets are balanced 88.4% (3 years), 79.2% (5 years), 78.6% (7 years), and 79.6%, 76.3%, 70.5% respectively. Callaghan et al. [16] determined the associations between individual metabolic syndrome components and peripheral neuropathy. The authors used multivariable models to assess for associations (LR and classification tree). The results showed a need for effective interventions that target these metabolic factors to prevent or treat peripheral neuropathy. In the clinical area, Sanchez et al. [17] analyzed the performance of eight different variable selection methods, of which stand out: regression-based methods and tree-based methods. The prediction performance was measured using the area under the ROC curve of the model on the testing set. In conclusion, Boruta was the most accurate model with 79.6% of AUC. Another approach for feature selection is that proposed by Chen et al. [18], which presents an analysis of various features that are useful for the classification data by implementing Machine Learning models such as Linear Discriminant Analysis (LDA), SVM, Random Forest and K-Nearest Neighbor (KNN). The research showed that by combining feature selection methods with the aforementioned models, Random Forest achieves a better performance in all experimental groups. Rghioui et al. [19] proposed and developed a 5G architecture for continuous monitoring of diabetic patients using machine learning algorithms (Naïve Bayes, ZeroR, OneR, LR, RF and Sequential Minimal Optimization [SMO]) for data classification. Finally, the SMO algorithm exhibited an excellent classification with the highest accuracy of 99.66%, giving a superior classification compared to other algorithms. Chen et al. [20], evaluate an automated software tool for nerve fibre detection and quantification in corneal confocal microscopy (CCM) images. The evaluation of the model used 888 images from 176 subjects. Then a ROC analysis was made, obtaining an AUC of about 0.77 and 72% sensitivity-specificity at the equal error rate point. Additionally, Pourhamidi et al. [21], compare the diagnostic usefulness of tuning fork, monofilament, biothesiometer and skin biopsies in peripheral neuropathy in subjects with T2D. The authors conclude that the tuning fork was a relatively good method to identify DSPN cases in terms of sensitivity obtaining 46%, otherwise, the biothesiometer achieved 67%, also, an intraepidermial nerve fibre densitity showed 74% and specificity of 70% in detection of DSPN. Ultimately, concluding, that using a biothesiometer in clinical routine might be a sensitive method to detect large nerve fibre dysfunction.

In particular, T2D and complications have contributed to the burden of mortality and the suffering of a single patient. Medical care, treatment options, care needs, and associated cost are complicated by existing comorbidities and chronic conditions [22]. The significant problem lies in the difficulty that exists in the identification and early detection of undiagnosed DSPN. Additionally, the lack of non-invasive tools necessary in the public and private health institutions cause a late identification of factors associated with chronic diseases, which are strong contributors to the timely prevention, prediction, correct decision-making in the treatment provided to the patient and finally in the reduction cost. The main contribution of this paper focuses on identifying possible predictors of DSPN. The aim of feature selection is to find out which features are useful for the classification data and Random Forest is essential for classifying the subjects with this condition. Finally, this work provides a description and analysis for future research which could be of great help to the medical field.

The remainder of the paper is organized as follows: Section 2 presents the dataset description and methodology to study the relationship of features to classify patients with and without distal symmetric polyneuropathy. The Section 3, shows the experiments performed using Boruta and Random Forest and additionally, the evaluation of the model with receiver operating characteristic curve (ROC curve), AUC, sensitivity and specificity. Finally, a discussion and conclusion of the results are presented in Section 4.

## 2. Materials and Methods

The methodology proposed in this work is contained in four main stages. A data pre-processing step was performed to avoid any problem related to missing data or outliers that could affect the later stages. Then, a feature selection method is presented, which was carried out using Boruta [23], it can select sample group relevant features effectively. To evaluate how well the selected features can classify the sample, one algorithm was applied: Random Forest (RF). Finally, all the models were evaluated on the basis of different parameters: sensibility, specificity, and area under the curve (AUC).

### 2.1. Data Description

The dataset for this study was acquired from “Unidad de Investigación Médica en Bioquímica, Centro Médico Nacional Siglo XXI, IMSS”, with the information of Mexican patients. All Mexican patients signed an informed consent letter and the protocol meets the Helsinki criteria which were approved by the Ethics Committee of Instituto Mexicano del Seguro Social under the number R-2011-785-018. The dataset is comprised of 32 features listed in Table 1 which includes clinical, para-clinical, and additional information of patients with T2D (HbA1c, GFR, and drug treatment). The total number of patients used for this work is 140 of which 70 corresponds to diabetic patients without any microvascular complications (controls) and 70 to diabetic patients with Distal Symmetric Polyneuropathy (cases). It is important to mention that the diagnosis of DSPN was made under family history and clinical evaluation. The age of the patients are between 31 and 84 years old, 65 are males while 75 are female.

### 2.2. Data Pre-Processing

For the pre-processing stage, several features were eliminated from the original dataset (ID, retinopathy and nephropathy cases) because the information was not relevant for this work. Furthermore, there were some missing values (GFR, SBP, DBP, SBPU, DBPU) represented as NA and were imputed with the value calculated using the mean of the non-missing observations. Finally, the 32 features were normalized through the standard score, where $x_{i}$ represents the original value, $\bar{x}$ is the mean of the sample, and *s*, is the standard deviation value of the feature (sample). The aim of this stage is to transform the data to a normal distribution with mean 0 and standard deviation 1.

(1) $$z_{i}=\frac{x_{i}-\bar{x}}{s}$$

### 2.3. Boruta Feature Selection

Boruta is a feature selection method based on Random Forest. This algorithm consist of the following steps [23]:

- Generate copies of all variables.
- Shuffle the added variables (attributes) to eliminate their correlations with the response.
- A RF classifier is executed and gather the Z scores computed.
- Find the maximum Z score among shadow attributes (MZSA) and then assign a value to each attribute that scored better than MZSA.
- For each attribute of undetermined importance, a two-sided equality test should be performed with the MZSA.
- Consider the attributes which have importance significantly lower that MZSA as unimportant and permanently remove them from the system.
- Consider the attributes which have importance significantly higher than MZSA as important.
- Eliminate all shadow attributes.

The benefits of Boruta are to decide the significance of a variable and to assist the statistical selection of important variables [18]. In other words, Boruta compares the performance of numerous models with successive variables replaced by shadow features. Then, this features are compared to each original feature that consistently outperform the collective maximum of the shadow versions. Finally, the features are classified as important and unimportant considering the MZSA value [24].

### 2.4. Classification Method

The supervised Machine Learning algorithm is selected to perform binary classification of the dataset described above. To predict whether a patient is diabetic and has DSPN or diabetic without this complication, we have used Random Forest (RF).

#### Random Forest

In 2001 Breiman et al. [25] developed the popular RF machine learning algorithm, which is based on classification and regression trees. The benefits of using RF are that this algorithm provides higher accuracy compared to a single decision tree, it has the ability to handle datasets with a large number of predictor variables, and can be used for variable selection [26]. It is important to note that RF has been successful in various areas, including the classification and identification of the most important variables in ecology [27], the diagnosis and prognostic for breast cancer [28], the applications in genomic data [29], among others. The RF algorithm consists of the following steps [30]:

- Fist, the dataset $D_{1}$ having *m* x *n* is given. Then, a new dataset $D_{2}$ is created from $D_{1}$ by sampling and eliminating a third part of the row data.
- The RF model is trained to generate a new dataset from the reduced samples, estimating the unbiased error.
- At each node point, the column $n_{1}$ is selected from the total *n* columns.
- Finally, several trees are growing and the final prediction is calculated based on individual decisions to obtain the best classification accuracy.

### 2.5. Validation

The performance of the proposed method is evaluated by comparing the two models with different metrics. In this study, the patients were labeled with 0, which are those who have development diabetes, and the case patients were labeled with 1, which are those who have DSPN. These outputs are represented within a confusion matrix, which is a table that shows the differences between the predicted classes for a set of labeled (reference) examples. It contains True Positives TP, True Negatives TN, False Positives FP and False Negatives FN. The diagonal is associated to the observations that are correctly classified.

- TP: number of instances that are positive and are correctly identified.
- TN: negative cases that are negative and classified as negative.
- FP: defined by the negative instances that are incorrectly classified as positive cases.
- FN: number of positive cases that are misclassified as negative.

There are many metrics that can be estimated to measure the performance of the models. However, in this work it was used to calculate two metrics: sensitivity and specificity.

Sensitivity corresponds to the accuracy of positive examples, it refers to how many examples of the positive classes were labeled correctly. This can be calculated with Equation (2).

(2) $$Sensitivity=\frac{TP}{TP+FN}$$

The specificity corresponds to the conditional probability of true negatives given a secondary class, which means that it approximates the probability of the negative label being true. It is represented by Equation (3).

(3) $$Specificity=\frac{TN}{TN+FP}$$

Also, a statistical analysis was conducted, obtaining the Receiver Operating Characteristic Curve (ROC), known as Area Under the Curve (AUC). Mostly, the quality of the algorithms (models) is evaluated by analyzing how well it performs on a test data [31]. The AUC, widely used to measure the performance in the supervised classification, is based on the relationship between the sensitivity and specificity [32]. The ROC analysis has become a popular method for evaluating medical diagnosis systems. This metric can discriminate two patient states, in this case with or without DSPN [33]. Furthermore, it has an important statistical property, in practice the value of AUC varies between 0.5 and 1, indicating the probability that the classifier will rank a randomly chosen positive instance higher than a negative instance.

All the methodology was performed using R (version 4.0.3), which is a free software environment for statistical computing and graphics [34]. The libraries used were Boruta (version 7.0) [23], caret (version 6.0-86) [35], and MLeval (version 0.3) [36].

## 3. Experiments and Results

**Figure 1:**
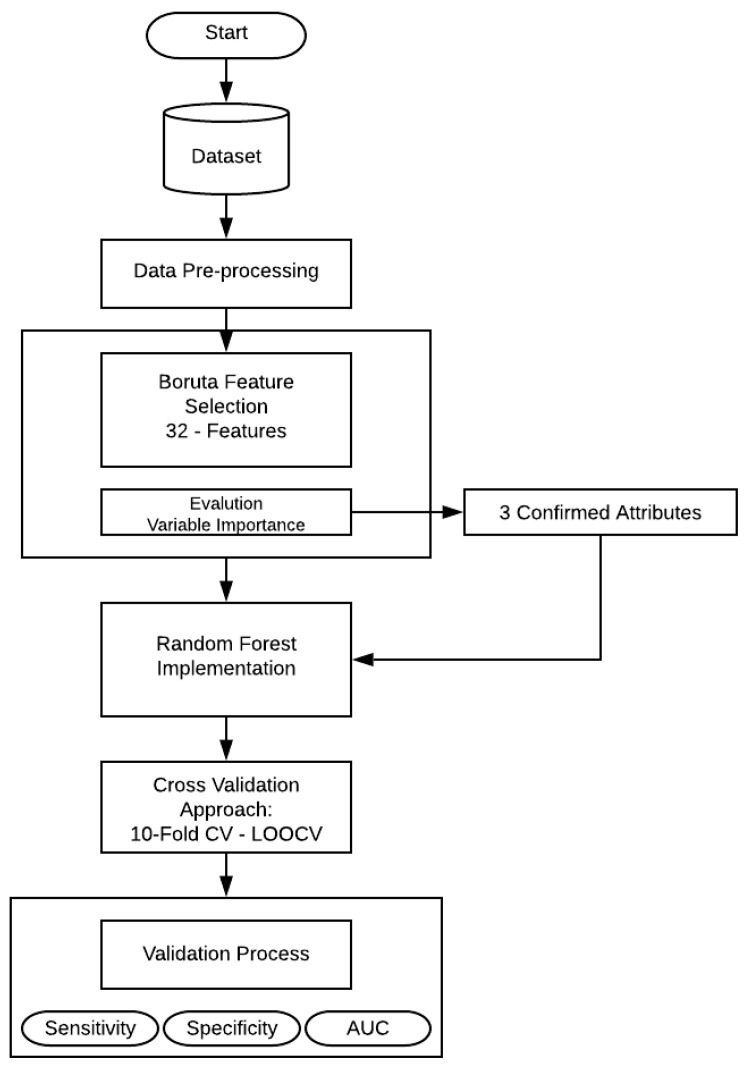
Flowchart of the proposed methodology.

This section presents the experiments and the results obtained in the development of this research. The entire structure of the proposed methodology can be shown in Figure 1.

First, a feature selection was performed using Boruta algorithm, that is implemented to finding all relevant attributes. Then the selected variables (confirmed features) serve as the input variables for RF technique. In the present work, RF create numerous independent decision trees, combining many decision trees produce more accurate classifications. Moreover, it includes calculation of variable importance and measures of similarity of data. The principal approach was to include all the 32 features and make an evaluation of the model, as mentioned in Table 2. RF needs some additional information (hyper-parameters) that should be considered. In this case, mtry, the number of random variables as candidates in each branch. The aim of this step is to choose a subset of predictors randomly and then splitting each node of trees with the best subset of all predictors. Secondly, with the aim of feature selection method, the RF model was trained and evaluated by statistical metrics.

Table 2 shows the hyper-parameters of the RF models, that were trained to classify DSPN patients.

RF can be used for solving regression or classification problems. In this case, the *y* variable is a factor value that applies to the classification. Then, the numbers of trees (ntree) are 500, which means that 500 trees were grown.The third parameter is the number of predictors sampled for splitting at each node (mtry), for the model with 32 features the mtry was 2, 17 and 32, and for the model with three features were 2 and 3 predictors.

The dataset used for the development of the methodology contains 32 features that include 140 observations of 32 variables and one output class. A total of 140 subjects were included in this study and were classified into a non-DSPN group and a DSPN group. To evaluate the performance of the RF model, a K-Fold Cross-Validation (CV) was performed, Leave One Out Cross Validation (LOOCV) and calculated its sensitivity, specificity, confusion matrix, ROC curve, and AUC value.

To test the efficiency of the classification, K-Fold CV and LOOCV were used, these are the most widely used methods for predictor evaluation. The K-Fold CV is repeated for *K* times, and the results can be calculated with a mean value and with a standard error rate. One of the advantages of performing a K-Fold CV is that with a small dataset, it could acquire a relatively stable evaluation of the model [37]. Secondly, LOOCV is a special case of K-Fold CV, where LOOCV divides the dataset into the number of instances in the dataset. Thus, LOOCV is applied for each instance, using all other instances as a training set and using the selected instance as a single test set [38]. For example, “140” subjects were collected. Firstly, “139” subjects as training set executed RF, then we had the first result and the “140” subject is back to the complete data, after, the second person as a testing set, leaving “139” patients as a training set, and we obtain the result of each subject. In this case, 10-Fold CV was used. Therefore, the training dataset was divided into 10 subsets, with one subset for the validation. This process was repeated three times.

The Table 3 shows the performance of the RF model with 10 -Fold CV joined by LOOCV. This approach is used for doing a test that guarantees a greater number of tests without the computational cost. Performing more combinations allows evaluating the models in different situations to try to avoid the over fitting. For real-word datasets Kohavi [39] recommends 10-Fold CV, and LOOCV estimates the generalization ability of a predictive model, and the computational cost can also be high for a large dataset [40]. The parameter mtry is the number of variables tried at each split, where the final value used for the optimal model was 17. The results of this classification demonstrate that sensitivity values oscillate from 63.80% to 64.91%. Nevertheless, in terms of AUC, the best model was 32, because it obtained 65.71% of AUC, representing a fair model performance.

Table 4 presents the confusion matrix of the model with the best outcome based on the performance measures. The performance of the classifier can be evaluated visually, and to determine which classes are highlighted. The correct predictions are located in the diagonal of the table, and the off-diagonal correspond to the incorrectly classified observations.

Table 4 gives a brief description of the classification error in each class used in RF.

**Figure 2:**
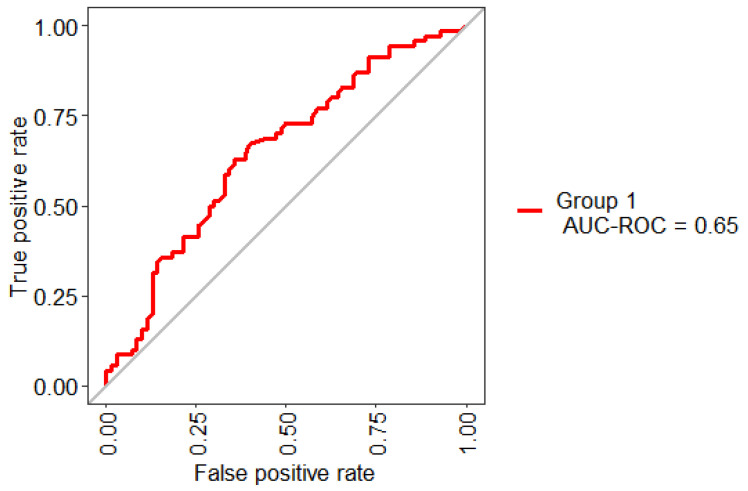
Receiving operating characteristic (ROC) curve obtained for the model based on the total set of features.

Figure 2 presents the ROC Curve obtained based on the performance of the RF using 10-Folds CV and LOOCV with 32 features. This model shows an AUC value of 65%. In general, an AUC of 0.5 indicates no discrimination, it means that the result of Figure 2 (65%) can model the problem or has the ability to diagnose patients with or without the condition.

The belief that “the more the variables, better the performance” is no longer acceptable. The application of feature selection has been gaining popularity in the field of data mining [41] and, the clinical area is no exception. The prediction of clinical outcomes is a common medical information need that is particularly adept to the use of clinical datasets, making predictive clinical modeling a promising area of study in digital healthcare [17].

The microvascular complications of DT2, especially DSPN, and can result in significant increase in morbidity, chronic pain, foot ulcerations, amputations and mortality [42]. The delay in the diagnosis of DSPN makes it difficult to treat and the early intervention is essential to prevent the progression. Feature selection plays an important role since it will obtain the relevant features to classify and identify the subjects with this condition.

**Figure 3:**
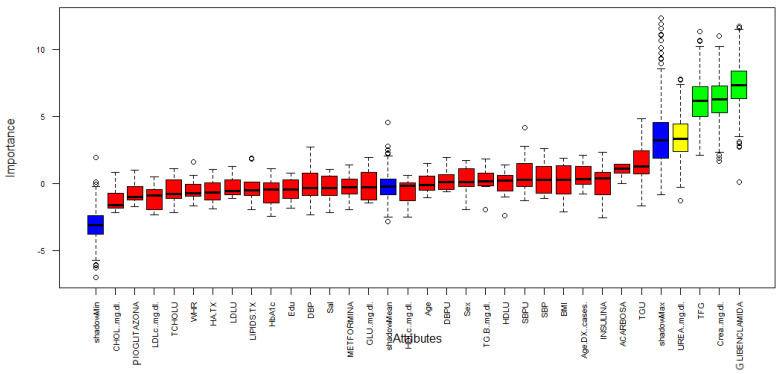
Variable Importance.

Figure 3 describes the importance for each variable of the dataset. Boruta performed 499 iterations. In this process, three attributes was confirmed important: GFR, Creatinine and Glibenclamide (green boxplot represent confirmed attributes), one attribute was confirmed as tentative: Urea (yellow bloxplot), 28 attributes was confirmed unimportant (red boxplot) and the blue bloxplots correspond to minimal, average and maximum Z score of a shadow attribute.

Table 5 presents a summary of the three features that are relevant for the classification of DSPN.

**Figure 4:**
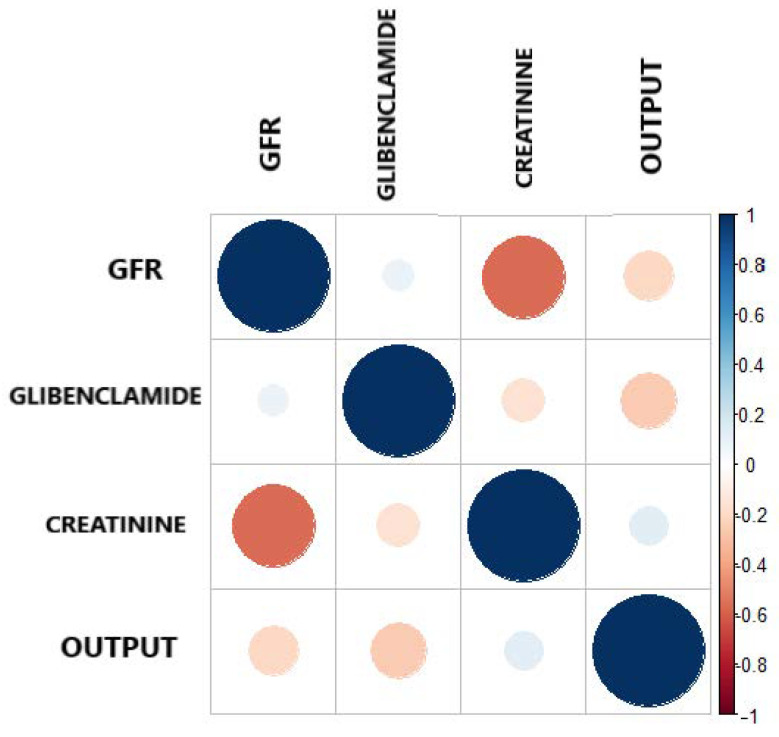
Correlation plot between the output and the three most important features.

Figure 4 and Table 6 present the Pearson correlation of the variables used in the model, playing an important role in descriptive analysis. Taking into consideration, the correlation ranges from −1 to 1. Figure 4 shows the different values of the correlation coefficient of the variables. The correlogram shows correlation for all pais of variables, the more intense colors are for more extreme correlations.

On the other hand, reaffirming the criteria, that correlation coefficient is a simple statistical measure of relationship between the variable dependent and one or more independent variables. The results show that the three features selected by Boruta, independently show a correlation as shown in Table 6, all having a negative correlation with respect to the output. This indicates that GFR, Glibenclamide and Creatinine have a low negative correlation, but they support the model in a joint way.

Table 7 shows the most accurate model using only three features, implementing Boruta as feature selection model joined by RF applying 10-Folds CV and LOOCV. The results of the classification demonstrate that AUC values oscillate from 66.05% to 67.01%. Although, the sensitivity and specificity have the same values, the AUC includes all the possible decision thresholds offering a more complete assessment.

The confusion matrix of the model with the best outcome is presented in Table 8. The final model achieved a sensitivity of 55.71% and specificity of 65.71%, with a minimum classification error.

**Figure 5:**
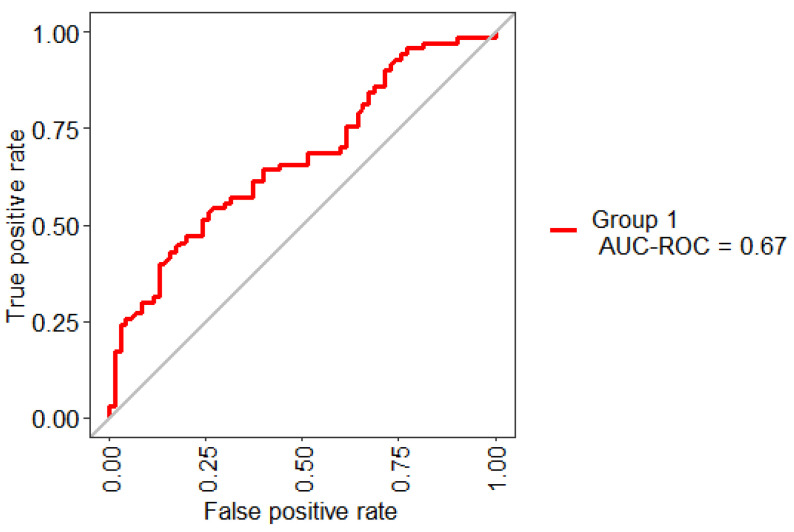
ROC curve obtained for the model based on the selected three features.

Figure 5 shows the statistical model performance obtained by implementing Boruta + RF with 10-Folds CV and LOOCV. This method showed an AUC of 67% and an Out-Of-Bag (OOB) error of 37.14%; this metric is an unbiased estimated of the true prediction error.

**Figure 6:**
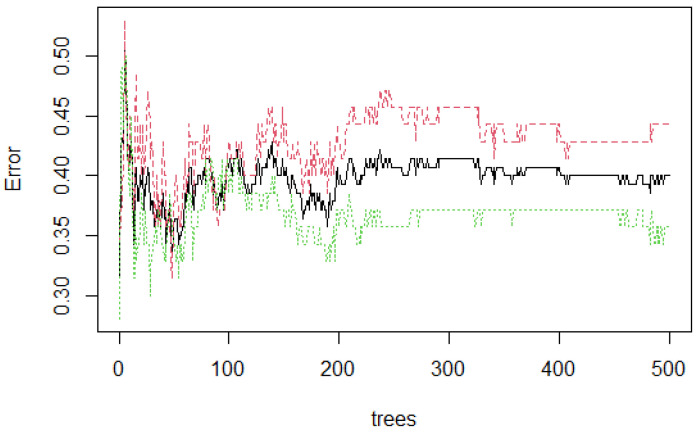
Random Forest (RF) stabilization.

Figure 6 presents the stabilization of the model developed with three features. As the number of trees grow, the stabilization occurs when 500 were reached. The error rate is 0.40, which means that 0.60 of the samples were correctly classified by RF.

## 4. Discussion and Conclusions

In this section, the discussion and conclusion of the results obtained for the different stages applied in this work are presented. Initially, the dataset contains 47 features. Nevertheless, a pre-processing stage was performed, removing the features that are not relevant to this work. Thus, the final dataset was comprised of 32 features listed in Table 1. The total number of patients used is 140 of which 70 (controls) corresponds to diabetic patients and the rest (70 cases) corresponds to diabetic patients with DSPN. Then, all the variables (features) were normalized through the standard score.

The classification algorithm of Random Forest was selected. This algorithm has been extensively used in bioinformatics, genetics, clinical, and other areas, and has been demonstrated to be an effective modeling technique [43]. For this purpose, the 32 features were submitted to the modeling using LOOCV and 10-Fold methods. A peculiarity for the evaluation in selection problems, is a topic known as CV and has been described for decades [44]. As mentioned earlier, proposals and evaluations have been made mixing the previous techniques [45,46,47], as well as a Multifold CV (MCV) and r-fold-CV proposal described by Zhang [44], which analyzes the essential test method as a bootstrap making a resampling of the observations. Retaking Zhang’s approach (bootstrap method and MCV) it is proposed LOOCV and 10-Fold CV. First, LOOCV is an expensive method, likewise, 10-fold CV wastes 10% of the data. However, using the proposed method, where LOOCV does not waste data, that means that 10% of lost data is recovered in each fold by using LOOCV.

Besides, sensitivity, specificity, ROC Curve, and AUC were used to verify the performance of the algorithm. Here, the AUC and ROC Curve are widely used in biomedical research literature [48,49,50], because it is the way to demonstrate the performance of a medical diagnostic test to detect or classify if a subject has the disease [51]. An ideal test has an AUC of 1, nevertheless, a value < 0.5 is above the diagonal in the ROC Curve, so, it is considered to have a reasonable discriminating ability, and it is useful to describe and model the problem [52].

According to the Fawcett criterion, the interpretation of the AUC values is as follows [53]: (0.5, 0.6) = bad test, (0.6, 0.75) = regular test, (0.75, 0.9) = good test, (0.9, 0.97) = very good test, and (0.97, 1) = excellent test. These values were used to interpret the performance of the models.

All the AUCs oscillate from 63.80% to 65.71% (Table 3) indicating that the models can model the problem. In other words, they have the ability to diagnose or classify patients with DSPN.

However, the main contribution of the present work focuses on identifying possible predictors of DSPN. Feature selection has been used in various domains including genetics, biomedicine, and informatics [54,55]. The main idea of this technique is that there are irrelevant features in the dataset which may reduce the classification accuracy, then, choose a small subset of features. As the small subset is much smaller than the entire one, the computation time of subsequent analysis is reduced [56]. Boruta, is a wrapper algorithm that is based on building classification models to determine the importance of features. The three most important features were calculated, Glomerular Filtration Rate, Creatinine, and Glibenclamide. Now, these three features comprise a new dataset. Implementing Boruta + RF applying the approach of 10-Folds CV and LOOCV. Once the model has been built, it is important to measure the performance of the model, because it provides an unbiased estimate of errors. The prediction of the model was based on different evaluation criteria. Sensitivity provides the portion of positive instances that were correctly classified. Specificity, the portion of negative instances that were correctly classified, and ROC Curve, a plot of the sensitivity versus 1- specificity, this metric can be considered as the average value of the sensitivity for a test over all possible values of specificity or vice versa [57]. The model with three features achieved 67% of AUC, thus proving the importance of “feature selection”.

Secondly, neuropathy manifests in different ways. DSPN, the most common form of diabetic neuropathy, is a chronic, nerve-length-dependent, that affects at least one-third of persons with type 1 or type 2 diabetes [58]. To diagnose this condition, the clinical diagnosis and a physical examination focus on vascular and neurologic tests.

The study has demonstrated the relationship between DSPN and GFR + Creatinine + Glibenclamide. Initially, creatinine clearance is an important factor that affects wound healing in patients with neuropathic diabetic foot ulcers and is widely used to estimate GFR. Moreover, these patients have impaired kidney functions, increasing risk for poor wound healing and amputation [59]. Diabetic Nephropathy is a clinical syndrome characterized by a decline in GFR, and a high risk of cardiovascular morbidity and mortality [60].

DSPN is directly associated with diabetic retinopathy due to diabetic microvessel disease is implicated, not considered a risk factor but is part of the same physiopathological [5]. The study of Dyck et al. [61] demonstrates that graded severity of retinopathy is strongly associated with the severity of DSPN. Another serious complication is diabetic nephropathy, this is more common in subjects with retinopathy and the severity of nephropathy increased with severity of retinopathy, and also, DSPN is more frequent in subjects with nephropathy. At last, nephropathy is univariately closely linked to DSPN and retinopathy [62].

On the other hand, Glibenclamide, a medication used to treat T2D. However, this drug has secondary effects such as decrease intake, chronic renal failure, among others [63]. Even though, the use of Glibenclamide has been decreasing in many countries, it is still used in Mexico, and the relationship with DSPN is clear, which indicates a direct impact on diabetic patients who develop DSPN. As mentioned earlier, GFR has been a strong indicator to identify chronic kidney disease. Additionally, nephropathy is clinically detected if the following criteria are met: persistent albuminuria and diabetic retinopathy [64].

In conclusion, this paper focuses on identifying the predictors of DSPN based on a methodology contained in four main stages. The two main models presented in this work, were 32 - Features and 3 - Features. Statistically, both models are completely different, since the entire dataset was used in the first model, then Boura was used for feature selection with the purpose of having only those that really contribute to the model. Finally, it is shown that using this type of techniques, a statistically significant result is obtained. For the feature selection, Boruta has confirmed the three important attributes: GFR, creatinine, and glibenclamide. Then a classification stage was done using RF with a cross-validation approach. Afterward, the performance measures were calculated. Finally, the model with three features reached 67.01% of AUC, demonstrating the effectiveness of classifying DSPN with a lower number of features. Therefore, this allows us to conclude that DSPN is strongly associated with diabetic nephropathy and diabetic retinopathy based on the model developed. Also, a timely diagnosis, education of patients, and continuous medical care are required to minimize the long-term complications. In addition, it should be noted that this is a preliminary tool that can be of great support for specialists in the diagnosis of DSPN based on a non-invasive method and may improve their decision making in the management of diseases and therapy planning. In future works, it would be interesting to reproduce this analysis with the inclusion of nephropathy and retinopathy data, which probably allows increasing the performance. Furthermore, we would like to probe more Machine Learning algorithms with the aim to compare their performance with another approach of feature selection algorithms which expands the criterion. 

## Figures and Tables

**Table 1:** Features description.

Feature	Description	Possible Values
Education	Studies concluded by the patient	1 - Elementary School 2 - Secondary School 3 - Technical level 4 - High School 5 - Professional 6 - Postgraduate
Salary	Monthly income	1 - Less than $2000.00 2 - Between $2000.00 and $5000.00 3 - More than $5000.00
Sex	Patients sex	0 - Male 1 - Female
Age	Age in years	Numeric Integer
Age DX	Diagnosis age of diabetes	Numeric Integer
WHR	Waist Hip Ratio	Numeric
BMI	Body Mass Index	Numeric
Glucose	Blood glucose levels	Numeric
Urea	Waste product resulting from the breakdown of protein in the patient body. The test can provide important information about the kidney function	Numeric Integer
Creatinine	Waste product produced by muscles as part of regular daily activity. The test is used to see if the kidneys are working normally	Numeric
Cholesterol	Fat-like substance that is found in all cells of the patient body	Numeric
HDL	Stands for High Density Lipoprotein (corrected for medication)	Numeric
LDL	Stands of Low Density Lipoprotein (corrected for medication)	Numeric
Triglycerides	Type of fat found in the patient body	Numeric
TCHOLU	Total Cholesterol (uncorrected)	Numeric Integer
HDLU	High Density Lipoprotein (uncorrected)	Numeric Integer
LDLU	Low Density Lipoprotein (uncorrected)	Numeric Integer
TGU	Triglycerides (uncorrected)	Numeric Integer
SBP	Systolic Blood Pressure (corrected for medication)	Numeric Integer
DBP	Diastolic Blood Pressure (corrected for medication)	Numeric Integer
SBPU	Systolic Blood Pressure (uncorrected)	Numeric Integer
DBPU	Diastolic Blood Pressure (uncorrected)	Numeric Integer
HA-TX	Hypertension Treatment	0 - No 1 - Yes
Lipids TX	Lipids Treatment	0 - No 1 - Yes
HbA1c	Glycated Hemoglobin	Numeric
GFR	Glomerular Filtration Rate (blood test that checks how well the kidneys are working)	Numeric Integer
Glibenclamide	Drug Treatment	0 - No 1 - Yes
Metformin	Drug Treatment	0 - No 1 - Yes
Pioglitazone	Drug Treatment	0 - No 1 - Yes
Rosiglitazone	Drug Treatment	0 - No 1 - Yes
Acarbose	Drug Treatment	0 - No 1 - Yes
Insuline	Drug Treatment	0 - No 1 - Yes
Output	Neuropathy State	0 - No 1 - Yes

**Table 2:** Random Forest parameters.

Parameters	
Type of Random Forest (y):	Classification
Number of trees (ntree):	500
No. of variables tried at each split (mtry):	2, 3, 17 and 32

**Table 3:** Performance parameters for the model with 32 features.

mtry	Sensitivity	Specificity	AUC
2	63.80%	55.71%	61.42%
17	64.91%	62.85%	62.85%
32	64.27%	62.85%	65.71%

**Table 4:** Confusion Matrix-32 features.

	Reference			
		**0**	**1**	**Class. Error**
**Prediction**	**0**	43	27	0.3857
	**1**	26	44	0.3714

**Table 5:** Three key features identified by Boruta.

	Features
1	GFR
2	Creatinine
3	Glibenclamide

**Table 6:** Correlation Matrix.

	GFR	Glibenclamide	Creatinine	Output
**GFR**	**1.0**	0.0840	−0.5616	−0.2091
**Gliblenclamide**	0.0840	**1.0**	−0.1585	−0.2572
**Creatinine**	−0.5616	−0.1585	**1.0**	0.1219
**Output**	−0.2091	−0.2572	0.1219	**1.0**

**Table 7:** Performance parameters for the model with three features.

mtry	Sensitivity	Specificity	AUC
2	55.71%	65.71%	67.01%
3	55.71%	65.71%	66.05%

**Table 8:** Confusion Matrix—three features.

	Reference			
		**0**	**1**	**Class. Error**
**Prediction**	**0**	39	31	0.4428
	**1**	25	45	0.3571

## Data Availability

Not applicable.
